# A LASSO-Derived Risk Model for Subclinical CAC Progression in Asian Population With an Initial Score of Zero

**DOI:** 10.3389/fcvm.2020.619798

**Published:** 2021-01-15

**Authors:** Yun-Ju Wu, Guang-Yuan Mar, Ming-Ting Wu, Fu-Zong Wu

**Affiliations:** ^1^Department of Radiology, Kaohsiung Veterans General Hospital, Kaohsiung, Taiwan; ^2^Department of Health Care Administration, Chang Jung Christian University, Tainan, Taiwan; ^3^Physical Examination Center, Kaohsiung Veterans General Hospital, Kaohsiung, Taiwan; ^4^Faculty of Medicine, School of Medicine, National Yang-Ming University, Taipei, Taiwan; ^5^Department of Medical Imaging and Radiology, Shu-Zen Junior College of Medicine and Management, Kaohsiung, Taiwan

**Keywords:** zero score, CAC progression, subclinical atherosclerosis, prediction model, nomogram

## Abstract

**Background:** This study is aimed at developing a prediction nomogram for subclinical coronary atherosclerosis in an Asian population with baseline zero score, and to compare its discriminatory ability with Framingham risk score (FRS) and atherosclerotic cardiovascular disease (ASCVD) models.

**Methods:** Clinical characteristics, physical examination, and laboratory profiles of 830 subjects were retrospectively reviewed. Subclinical coronary atherosclerosis in term of Coronary artery calcification (CAC) progression was the primary endpoint. A nomogram was established based on a least absolute shrinkage and selection operator (LASSO)-derived logistic model. The discrimination and calibration ability of this nomogram was evaluated by Hosmer–Lemeshow test and calibration curves in the training and validation cohort.

**Results:** Of the 830 subjects with baseline zero score with the average follow-up period of 4.55 ± 2.42 year in the study, these subjects were randomly placed into the training set or validation set at a ratio of 2.8:1. These study results showed in the 612 subjects with baseline zero score, 145 (23.69%) subjects developed CAC progression in the training cohort (*N* = 612), while in the validation cohort (*N* = 218), 51 (23.39%) subjects developed CAC progression. This LASSO-derived nomogram included the following 10 predictors: “sex,” age,” “hypertension,” “smoking habit,” “Gamma-Glutamyl Transferase (GGT),” “C-reactive protein (CRP),” “high-density lipoprotein cholesterol (HDL-C),” “cholesterol,” “waist circumference,” and “follow-up period.” Compared with the FRS and ASCVD models, this LASSO-derived nomogram had higher diagnostic performance and lower Akaike information criterion (AIC) and Bayesian information criterion (BIC) value. The discriminative ability, as determined by the area under receiver operating characteristic curve was 0.780 (95% confidence interval: 0.731–0.829) in the training cohort and 0.836 (95% confidence interval: 0.761–0.911) in the validation cohort. Moreover, satisfactory calibration was confirmed by Hosmer–Lemeshow test with *P*-values of 0.654 and 0.979 in the training cohort and validation cohort.

**Conclusions:** This validated nomogram provided a useful predictive value for subclinical coronary atherosclerosis in subjects with baseline zero score, and could provide clinicians and patients with the primary preventive strategies timely in individual-based preventive cardiology.

## Introduction

Subclinical atherosclerosis is a chronic, progressive, and inflammatory disease of the arterial wall with a long-term asymptomatic phase (1–3). In recent years, non-invasive imaging modalities have been proposed to help early detect and monitor the burden of subclinical atherosclerosis. The introduction of several non-invasive imaging modalities has given the possibility to diagnose subclinical atherosclerosis easily in asymptomatic subjects, including carotid ultrasonography and coronary calcium assessment by computed tomography (CT). Coronary artery calcification (CAC) could be considered a surrogate marker of subclinical coronary atherosclerotic burden (2). Agatston score of zero is known to be a powerful negative cardiovascular event predictor with a long-term warranty period (“the power of zero”) (4–8). PESA study has demonstrated that male, age, high-density lipoprotein cholesterol (LDL-C), hemoglobin A1c (HbA1_c_), vascular cell adhesion molecule-1 (VCAM) and cystatin are significant biologic predictors associated with subclinical atherosclerotic lesions in Western asymptomatic population with low cardiovascular risk (9). Previous studies have investigated the risk factors associated with the warrant period of zero score in Asian population (8, 10, 11). To the best of our knowledge, no previous studies have evaluated early preventive models for predicting the risk of subclinical coronary atherosclerosis in term of CAC progression in Asian population with baseline zero score. Therefore, we aim to develop a LASSO (least absolute shrinkage and selection operator)-based risk model for the prediction of subclinical CAC progression with an initial score of zero in a hospital-based dataset, and to compare its discriminatory ability with other prediction models, such as Framingham risk score (FRS) and atherosclerotic cardiovascular disease (ASCVD) score.

## Methods

### Study Population and Baseline Characteristics

Eight hundred and thirty consecutive subjects were included in this study from April 2005 to December 2018 according to the inclusion criteria. The inclusion criteria for this study are as follows: (1) all subjects with medical check-ups underwent two consecutive scans (CAC scan and coronary CT angiography) during the follow-up period; (2) all subjects must meet the criteria of zero score in the baseline scan. Because we did not use any human subjects or personally identifiable records in our study, informed consent was waived. The study protocol was approved by the institutional review boards of Kaohsiung Veterans General Hospital in accordance with the Declaration of Helsinki (IRB: VGHKS19-CT6–02). Clinical characteristics, physical examination, and laboratory profiles were retrospectively obtained from the patients' electronic medical records and reviewed by a trained study coordinator. Laboratory profiles were performed at the same day as the baseline CT scans. Clinical demographic characteristics included age, gender, BMI, current smoking habit, pack-year, hypertension, diabetes mellitus and follow-up period collected by patients' electronic medical records or questionnaire. A physical examination was also conducted to collect data on body mass index (BMI), systolic blood pressure (SBP), diastolic blood pressure (DBP), body-fat percentage, and waist circumference. Laboratory profiles were collected to obtain biochemical variables, including uric acid, Gamma-Glutamyl Transferase (GGT), fasting glucose, hemoglobin A1c (HbA1c), C-reactive protein (CRP), low-density lipoprotein cholesterol (LDL-C), high-density lipoprotein cholesterol (HDL-C), total cholesterol, and triglycerides level. Hypertension was defined as systolic blood pressure (SBP) > 140 mmHg, diastolic blood pressure (DBP) >90 mmHg, or subjects with anti-hypertensive medications. Diabetes mellitus was diagnosed in subjects with additional oral anti-diabetic or insulin medications. Framingham risk score (%) in the first round, CAD-RADS categories in the first and final round, and CAC score in the final round were also recorded.

### CT Imaging Acquisition

All subjects retrospectively underwent two consecutive scans in the first round and final round during the mean follow-up period of 4.55 ± 2.42 years. In brief, a non-contrast CAC scan was performed before the cardiac CT angiography on a 256 × 0.625-mm detector row CT system (Revolution CT, GE Healthcare, Milwaukee, USA) or a 64 × 0.5-mm detector row CT system (Aquilion 64; Toshiba Medical Systems). CT acquisition protocol includes two sequential acquisitions.

First, a non-contrast CAC scan was performed with the following acquisition parameters: fixed tube voltage 120-kVp with reconstructed at 3 mm slice thickness. Secondly, a prospectively ECG-triggered cardiac CT angiography (CTA) was performed with the following parameters: fixed tube voltage of 120 kV, tube current modulation (mA modulation). CAC scoring was performed using the Agatston method with GE AW analysis software (12). Prior to cardiac CT angiography imaging, oral beta-blockers (metoprolol 100 mg) and sublingual nitroglycerin (nitrostat one tablet, 0.6 mg) were administered to all subjects without contraindication if the heart rate exceeded 65 beats per min. Intravenous contrast (Iopamidol 370) was administered at 5 mL/second followed by 40 mL 0.9% saline flush. Images were acquired and reconstructed at diastole (75–81% of the R-R interval) or at systole (37–43% of the R-R interval). All CT scans were reported by accredited cardiac radiologists. The severity of obstructive CAD with standardized reporting of individual segmental coronary stenosis was reported according to Coronary Artery Disease Reporting and Data System (CAD-RADS™), published in 2016 by the Society of Cardiovascular Computed Tomography (SCCT) (13).

### Three Prediction Models for Subclinical CAC Progression

#### LASSO-Derived Prediction Model

We extracted 10 features through lasso regression to construct the new prediction model with the optimal value of lambda that minimizes the cross-validation error, and compares its prediction accuracy and discriminatory ability with other different prediction models, such as FRS model and ASCVD model. In addition, we evaluated the discriminatory ability of different prediction models by using c-statistic, Akaike information criterion (AIC) and Bayesian information criterion (BIC). Higher c-statistic and lower AIC and BIC values were considered to indicate a more discriminatory model. Previous literature reviews have shown that original purpose of FRS and ASCVD models in 10-year cardiovascular event prediction (14, 15). However, there are some scientific merits of FRS and ASCVD models in subclinical atherosclerosis prediction according to recent studies (16, 17).

#### Framingham Risk Score (FRS) Model

FRS is the scoring system that is most commonly used to predict the 10-year cardiovascular events. The components of the FRS include age, sex, total cholesterol, HDL-C, systolic BP, DM, and smoking habit. A total FRS score was calculated for each eligible subject according to the algorithm developed by D'Agostini et al. (14). Current clinical guidelines recommend categorizing asymptomatic individuals into low (FRS <10%), intermediate (10–20%), and high-risk subgroups (> 20%) for risk stratification.

#### Atherosclerotic Cardiovascular Disease (ASCVD) Model

The ASCVD score includes predictors as age, sex, race, smoking habit, systolic blood pressure, diastolic blood pressure, and diabetes for prediction 10-year fatal outcome in individuals aged 40 to 65 years (15). Individuals were classified into low-risk (ASCVD <7.5%), and high-risk (ASCVD≧7.5%) subgroups for ASCVD risk stratification (18).

### Statistical Analysis

The clinical characteristics and demographic profiles of the subjects in the training and validation cohorts were compared by Student *t*-test for continuous variables and chi-square test/Fisher exact text for categorical variables. The primary study outcome was to develop a LASSO-based prediction nomogram (Optimal lambda selection) for CAC progression in Asian population with baseline zero-score (19). The multivariable logistic regression model was used to estimate the odds ratio (OR) and 95% CI. We evaluate and compare the discriminatory ability of three predictive models by using the c-statistic (area under the ROC curve, AUC), Bayesian information criterion (BIC) and Akaike information criterion (AIC). Higher c-statistic and lower AIC/BIC values were considered to indicate a more discriminatory model (20). The values of the c-statistic range from 0.5 (no ability to discriminate) to 1.0 (full ability to discriminate). Calibration was assessed by the Hosmer–Lemeshow goodness-of-fit statistic and by calibration graphs plotting predicted CAC progression against the observed rates in deciles of predicted risk (21). A nomogram was established based on the LASSO-derived parameters in the training cohort. The statistical significance for all tests was set at *P* < 0.05. All statistical analyses were performed using SPSS 22.0 for Windows (SPSS Inc., Chicago, IL) and Stata version 13.0 (Stata Corp, College Station, TX, USA).

## Results

### The Study Population Characteristics

Of the 830 subjects with baseline zero score in our study, of whom 555 were men and 275 were women, 196 had CAC progression events and 634 did not have the events.

The prevalence of CAC progression in the total study cohort was 23.61%.

**Figure 1:**
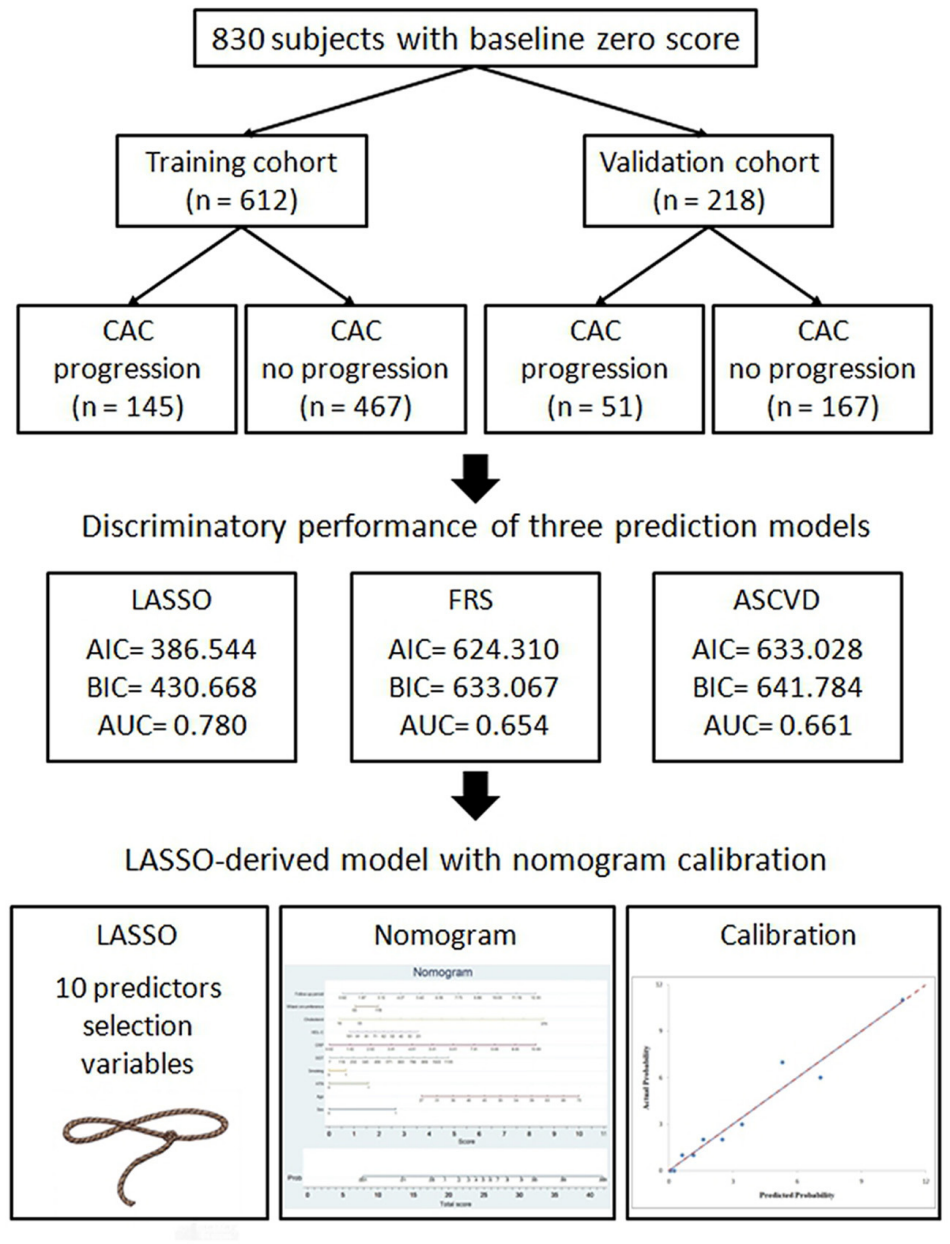
Flow chart of patient selection for the training and validation cohorts.

In the study cohort of 830 subjects, about 17.2 subjects have non-calcified plaques in the baseline scan. In the final round, about 41.9% subjects have non-calcified or calcified plaques formation during follow up period of 4.55 ± 2.42 year shown in Table 1. These subjects were randomly placed into the training set or validation set at a ratio of 2.8:1. 612 and 218 subjects with baseline zero score were included in the training and validation cohorts, respectively, shown in Figure 1. Table 1 summarizes the subjects' characteristics in the training and validation cohort. Our study included 830 subjects with baseline zero score in this study cohort. The basic clinical characteristics for the training cohort (612 subjects, mean age 51.26 ± 8.26, 66% male) and the validation cohort (218 subjects, mean age 50.90 ± 8.19, 69.3% male) are list in Table 1. In the training cohort, 145 subjects (23.69%) developed CAC progression, while in the validation cohort, 51 subjects (23.39%) developed CAC progression. There were no significant differences between the two groups (the train cohort and the validation cohort) in terms of all parameters of clinical characteristics, physical examination, and laboratory profiles.

**Table 1:** Clinical features of the training and validation sets.

	**Total patient cohort (*n* = 830)**	**Training set (*n* = 612)**	**Validation set (*n* = 218)**	***P*-value**
Age (years)	51.17 ± 8.24	51.26 ± 8.26	50.90 ± 8.19	0.576
Sex, *n* (%)				0.381
Male	555 (66.9%)	404 (66%)	151 (69.3%)	
Female	275 (33.1%)	208 (34%)	67 (30.7%)	
BMI (kg/m^2^)	24.72 ± 3.42	24.83 ± 3.40	24.41 ± 3.44	0.120
SBP (mmHg)	123.90 ± 16.99	124.23 ± 17.05	122.98 ± 16.84	0.355
DBP (mmHg)	78.13 ± 11.14	78.33 ± 11.14	77.58 ± 11.16	0.403
Hypertension, *n* (%)	245 (29.5%)	188 (30.7%)	57 (26.2%)	0.185
Smoking, *n* (%)	270 (32.5%)	194 (31.6%)	76 (34.9%)	0.303
DM, *n* (%)	104 (12.5%)	82 (13.4%)	22 (10.1%)	0.213
Uric acid (mg/dL)	6.37 ± 2.32	6.37 ± 2.52	6.39 ± 1.64	0.907
GGT (U/L)	42.18 ± 77.92	42.11 ± 72.26	42.36 ± 92.07	0.973
Fasting glucose (mg/dL)	99.85 ± 22.43	100.30 ± 22.76	98.57 ± 21.46	0.334
CRP (mg/dL)	0.21 ± 0.43	0.21 ± 0.48	0.18 ± 0.23	0.383
LDL-C (mg/dL)	116.29 ± 29.12	116.31 ± 29.22	116.25 ± 28.90	0.981
HDL-C (mg/dL)	47.16 ± 13.27	47.41 ± 13.33	46.47 ± 13.11	0.377
Cholesterol (mg/dL)	205.96 ± 37.48	206.76 ± 37.24	203.70 ± 38.15	0.306
Triglycerides (mg/dL)	151.98 ± 102.11	153.05 ± 100.21	148.96 ± 107.46	0.615
HbA1c (%)	5.88 ± 0.79	5.90 ± 0.83	5.83 ± 0.67	0.295
Body fat percentage (%)	23.97 ± 5.93	24.20 ± 5.82	23.34 ± 6.19	0.097
Waist circumference (cm)	85.70 ± 9.31	85.98 ± 9.29	84.93 ± 9.36	0.200
Follow-up period (year)	4.55 ± 2.42	4.59 ± 2.42	4.43 ± 2.41	0.416
First CAD-RADS categories (%)				0.260
0	682 (82.2%)	508 (84%)	174 (80%)	
≧ 1	143 (17.2%)	100 (16%)	43 (20%)	
Final CAD-RADS categories (%)				0.668
0	480 (58.1%)	356 (58.6%)	124 (56.9%)	
≧ 1	346 (41.9%)	252 (41.4%)	94 (43.1%)	
Framingham risk score (%)	12.79 ± 8.90	12.94 ± 9.00	12.40 ± 8.62	0.452
CAC score in the final round	5.17 ± 21.41	5.56 ± 23.59	4.05 ± 13.47	0.370

*BMI, body mass index; SBP, systolic blood pressure; DBP, diastolic blood pressure; DM, diabetes mellitus; GGT, gamma-glutamyl transferase; CRP, c-reactive protein; LDL-C, low-density lipoprotein cholesterol; HDL-C, high-density lipoprotein cholesterol; HbA1c, hemoglobin A1c; CAD-RADS, the coronary artery disease - reporting and data system; CAC, coronary artery calcification*.

### LASSO-Derived Predictor for Subclinical CAC Progression

We conducted logistic regression with the least absolute shrinkage and selection operator (LASSO) penalization to help reduce the dimensions of feature selection through a 10-fold cross validation for subclinical CAC progression prediction. Finally, ten of the original 20 variables were selected in the prediction model developing. The finial LASSO model with optimal lambda included the following 10 non-zero variables: “sex,” “age,” “hypertension,” “smoking habit,” “GGT,” “CRP,” “HDL-C,” “cholesterol,” “waist circumference,” and “follow-up period.” We carried out the multivariate analyses in the training cohort to establish the prediction model for subclinical CAC progression. Ten of the original 20 variables were included in the prediction model. The results of the multivariate logistic regression analysis are summarized in Table 2. The LASSO-derived prediction model including 10 selected variables also has showed its good performance in Table 2.

**Table 2:** LASSO-derived multivariable logistic regression for predicting CAC progression in subjects with baseline zero score.

	**Coefficient**	**OR**	**95% CI**	***P*-value**
**LASSO-selected 10 variable**				
Sex (%)	1.086	2.962	1.387–6.325	0.005
Age (year)	0.056	1.057	1.020–1.095	0.002
Hypertension (%)	0.639	1.896	1.099–3.269	0.021
Smoking (%)	0.276	1.318	0.738–2.353	0.350
GGT (U/L)	0.002	1.002	0.999–1.005	0.250
CRP (mg/dL)	0.336	1.399	0.923–2.123	0.114
HDL-C (mg/dL)	−0.014	0.986	0.961–1.011	0.260
Cholesterol (mg/dL)	0.009	1.009	1.002–1.016	0.009
Waist circumference (cm)	0.007	1.007	0.973–1.041	0.697
Follow-up period (year)	0.272	1.313	1.186–1.454	<0.001
**LASSO-derived model**				
Prediction model	5.256	191.667	47.062–780.597	<0.001

*LASSO, least absolute shrinkage and selection operator; CAC, coronary artery calcium; GGT, gamma-glutamyl transferase; CRP, c-reactive protein; HDL-C, high-density lipoprotein cholesterol; OR, odds ratio; CI, confidence interval*.

### Development of the Nomogram

**Figure 2:**
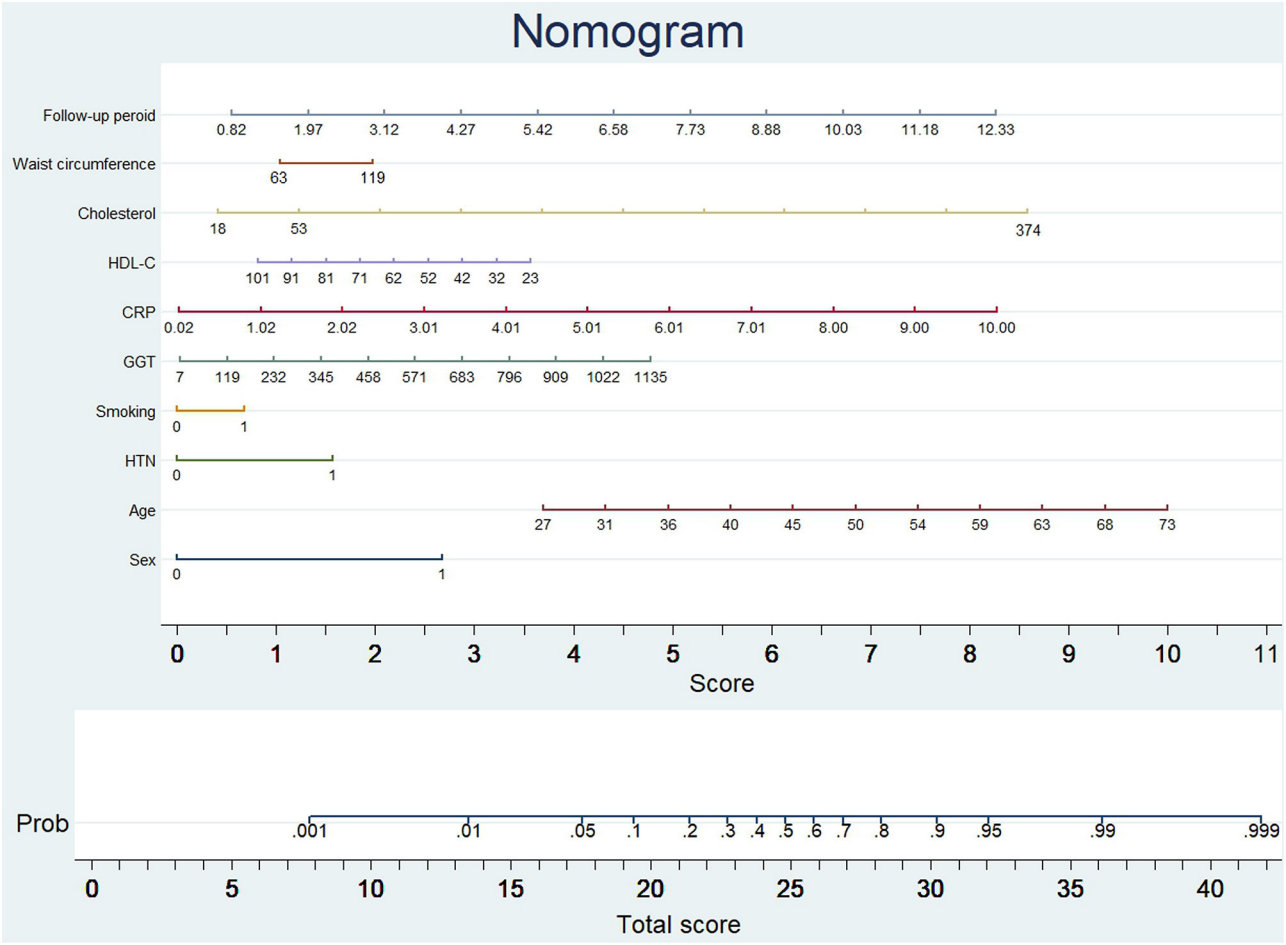
Nomogram to predict CAC progression in subjects with baseline zero score. (Figure was created by Stata 13, nomolog). To use the nomogram, an individual participant's value is located on each variable axis, and a line is drawn upward to determine the number of points received for each variable value. The sum of these numbers is located on the total points axis to determine the possibility of CAC progression.

The probability of subclinical CAC progression in the study training cohort with the baseline zero score according to the multivariable logistic regression model including ten potential predictive factors (sex, age, hypertension, smoking habit, GGT, CRP, HDL-C, cholesterol, waist circumference, and follow-up period). A nomogram was further generated to predict subclinical CAC progression based on the multivariable logistic regression results shown in Figure 2. By adding up these scores identified on the points scale for each parameter, we were easily able to draw a straight line down to establish the estimated individual probability score of subclinical CAC progression in the training cohort with baseline zero score. As an example to better explain the nomogram model, if the male subject is age of 58, Hypertension (+), smoking (−), GGT of 465 u/L, CRP of 9 mg/dl, HDL-C of 40 mmol/L, cholesterol of 374 mmol/L, waist circumference of 119 cm, and follow-up period of 8.8 year, the probability of subclinical CAC progression is estimated to be 99%.

### Internal and External Validation of the LASSO-Derived Nomogram

A 10-fold cross-validation method was applied to validate the nomogram model.

**Figure 3:**
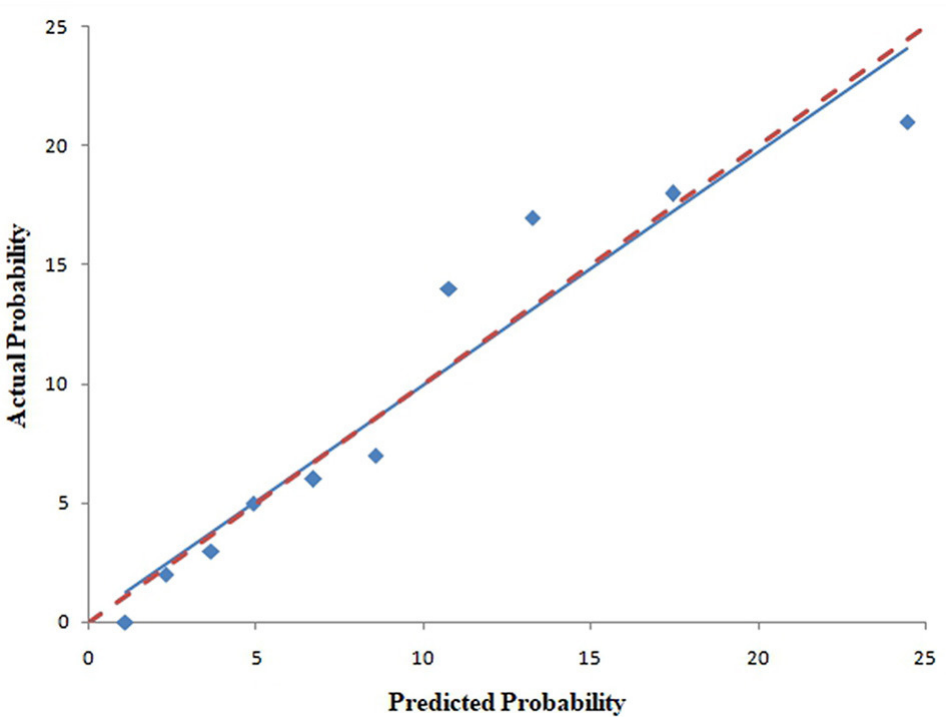
Calibration curves of the nomogram for predicting CAC progression from the training cohort. The Hosmer–Lemeshow test had a *p*-value of 0.654 in the training cohort.

**Figure 4:**
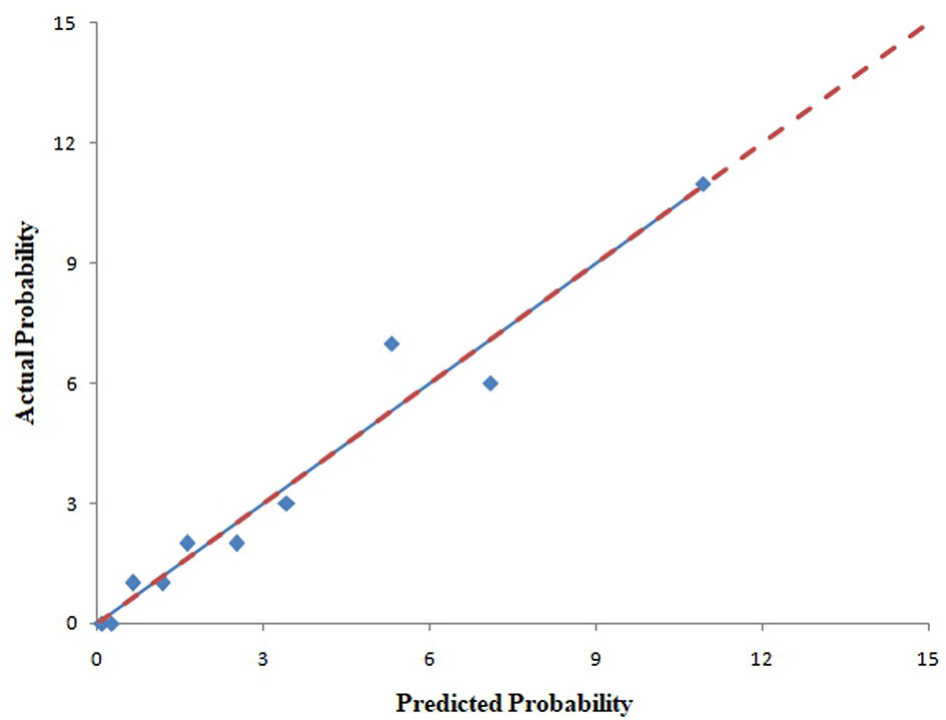
Calibration curves of the nomogram for predicting CAC progression from the validation cohort. The Hosmer–Lemeshow test had a *p*-value of 0.979 in the validation cohort.

The pooled area under ROC curve of the nomogram was 0.780 (95% confidence interval: 0.731–0.829) in the training cohort and 0.836 (95% confidence interval: 0.761–0.911) in the validation cohort. The ROC showed the resulting model had quite good discrimination in the training and validation cohorts. Good calibration was also demonstrated by non-statistical significance obtained in the Hosmer–Lemeshow test in both the training and validation cohorts (*p* = 0.654 in the training cohort; *p* = 0.979 in the validation cohort), as displayed by calibration curves shown in Figures 3, 4.

### Comparison of LASSO-Derived, FRS and ASCVD Models

Table 3 presents a summary of the discriminatory ability and diagnostic performance of the three prediction models, including LASSO-derived, FRS, and ASCVD models.

**Table 3:** Prediction performance of LASSO, FRS, and ASCVD models, *n* = 612.

	**AIC**	**BIC**	**AUC (95% CI)**	**Cut-point**	**Sensitivity**	**95% CI**	**Specificity**	**95% CI**	**+LR**	**95% CI**	**–LR**	**95% CI**	**+PV**	**95% CI**	**–PV**	**95% CI**
LASSO model	386.5443	430.6682	0.780 (0.731–0.829)	>0.2205	78.49	68.8–86.3	67.62	62.1–72.8	2.42	2.1–2.8	0.32	0.2–0.5	41.7	34.3–49.4	91.4	87.1–94.7
FRS model	624.3103	633.0672	0.654 (0.605–0.702)	>11.1	71.22	62.9–78.6	57.78	53.1–62.4	1.69	1.5–1.9	0.5	0.4–0.7	34.3	28.8–40.0	86.7	82.3–90.3
ASCVD model	633.0280	641.7848	0.661 (0.612–0.709)	>5.5	69.78	61.4–77.3	57.78	53.1–62.4	1.65	1.4–1.9	0.52	0.4–0.7	33.8	28.3–39.6	86.1	81.7–89.8

*LASSO, least absolute shrinkage and selection operator; FRS, Framingham risk score; ASCVD, atherosclerotic cardiovascular disease; AIC, Akaike information criterion; BIC, Bayesian information criterion; AUC, area under the ROC curve; CI, confidence interval; LR, likelihood ratio; PV, predictive value*.

In addition, the comparison and difference of three predictive models are summarized in Table 4. Our study result demonstrated that LASSO-based model has significantly superior discriminatory ability, higher c-statistic, and the lower AIC and BIC over other two predictive models. Compared with FRS and ASCVD model, the novel LASSO-derived nomogram model shows better diagnostic performance with an AUC of 0.780 (95% CI, 0.731 to 0.829) for detection subclinical CAC progression in Asian population with baseline zero score with balanced sensitivity (78.49%) and specificity (67.62%).

**Table 4:** Comparison of discriminatory ability in three predictive models.

	**Difference between areas**	**SE**	**95% CI**	***P*-value**
LASSO vs. FRS	0.1000	0.0276	0.0459–0.154	<0.001
LASSO vs. ASCVD	0.0952	0.0288	0.0387–0.152	0.001
FRS vs. ASCVD	0.0048	0.0088	−0.0125–0.022	0.587

*LASSO, least absolute shrinkage and selection operator; FRS, Framingham risk score; ASCVD, atherosclerotic cardiovascular disease; SE, standard error; CI, confidence interval*.

## Discussion

We built up and assessed a nomogram model for individually predicting subclinical CAC progression in subjects with baseline zero score. The predictive nomogram model incorporates clinical characteristics, physical examination and laboratory profiles for guiding individual subclinical coronary atherosclerosis prediction. To the best of our knowledge, this is a first predictive nomogram for subclinical CAC progression prediction in Asian population. In this study, we demonstrated three major findings. The first one is that we developed a LASSO-derived novel nomogram prediction model based on clinical characteristics, physical examination and laboratory profiles to predict subclinical atherosclerosis with baseline zero score, and demonstrated that it provides a good level of performance for predicting subclinical CAC progression in an Asian cohort. Second, compared with FRS and ASCVD model, the LASSO-derived model exhibited a significantly better discriminatory ability and lowest AIC and BIC. Third, the LASSO-derived risk prediction model exhibited good discrimination and calibration ability in the training and validation cohort.

In this study, we consecutively selected and analyzed 830 subjects with baseline zero score, which randomly divided into the training cohort and validation cohort. In the mean follow-up period of 4.55 ± 2.42 year, finally about 196 (23.61%) had CAC progression events in the study cohort. For subclinical CAC progression, LASSO-derived model with an optimal cut-off value of <0.2205 (probability score) may be an ideal screening tool to help rule out subclinical CAC progression within the 5 years of the warranty period in the middle-age Asian population with low to intermediate risk (sensitivity of 78.49%; specificity of 67.62%). Our study findings are consistent with a growing body of literature about the natural course of CAC progression in population with zero score (4–8). The evidences from previous studies have demonstrated that zero CAC score at the baseline scan could provide the 5 year- warranty period of beneficial effect on the future cardiac event in both Western and Asian asymptomatic population with low to intermediate cardiovascular risk. In addition, we developed and validated a new novel nomogram that integrated clinical characteristics, physical examination and laboratory profiles. This nomogram can more efficiently predict the subclinical CAC progression, compared with FRS or ASCVD model. Sarah et al. previously reported that CVHI (cardiovascular health index) score had most sensitive (94%) but least specific (14.9%) in identifying individuals with subclinical atherosclerosis assessed with non-invasive carotid intima-media thickness (CIMT) measurement, compared with FRS and MetS (metabolic syndrome) score (22). Our previous study has demonstrated that FRS score had poor to fair diagnostic performance for subclinical CAC progression prediction in individuals with baseline zero score (8). Compared with FRS and ASCVD model, the novel LASSO-derived nomogram model shows better performance with an AUC of 0.780 (95% CI, 0.731 to 0.829) for detection subclinical CAC progression in Asian population with baseline zero score with balanced sensitivity (78.49%) and specificity (67.62%). Our LASSO-derived model is feasible to predict subclinical CAC progression with high relative high sensitivity for rule out this clinical scenario in subjects with baseline zero score. Early detection of coronary atherosclerosis in its subclinical stage could impact on the primary prevention of cardiovascular events, and allow the prompt implementation of primary prevention strategies (1–3). The PESA study demonstrated that the high prevalence of subclinical atherosclerosis (44%) in term of the iliac-femoral district in asymptomatic middle-aged population (23, 24). Therefore, healthy lifestyle strategies such as lifelong attention to diet, exercise habit, smoking abstinence or statins treatment in the subgroup with CAC >100 through promoting patient-centered shared decision making are crucial for maintaining and prolong cardiovascular health and to slow the progression of coronary atherosclerosis in preventive cardiology (1–3, 25).

## Strengths and Limitations

The study has two main strengths. First, a strength of the present study was its longitudinal nature which allowed us to clearly identify the correct sequence of time events, identify changes over time, eliminate recall bias and provide insight into cause-and-effect relationships. Second, this study investigates on the unique Asian population cohort. However, there is no population-based study focusing on the prediction model for subclinical coronary atherosclerosis among Asian population. Therefore, this study could investigate risk factors of subclinical coronary arthrosclerosis associated with the racial difference.

There are some limitations in this study. First, this is a single-center retrospective study focused on Asian population. Therefore, the generalizability of the prediction model result to the western population is limited. Second, we did not investigate the clinical cardiovascular event for primary outcome analysis due to small sample size limitation and low to intermediate FRS risk (FRS% 12.79 ± 8.90, *N* = 830). Therefore, the cost-benefit analysis of predicting subclinical coronary atherosclerosis is still uncertain (26, 27). Subclinical coronary atherosclerosis has become a threatening public health issue in the world due to behavioral, environmental and genetic factors (28, 29). There is increasing trend in the USA that people died suddenly from cardiovascular events at low risk according to Framingham risk stratification (1, 2, 30). Therefore, to pay more attention on subclinical coronary atherosclerosis is mandatory in this age with high prevalence of subclinical atherosclerosis stage. Early detection with primary prevention such as health promotion with lifestyle behavior modification (diet, physical activity, stop smoking, etc.) is a very important way to slow or reverse the progression of subclinical coronary atherosclerosis (31, 32). Third, our relatively short follow-up period is a potential limitation. Therefore, longer follow-up studies are warranted to investigate the natural course of CAC progression in the 10-year period. Fourth in this study we aimed to investigate a specific form of subclinical coronary atherosclerosis in term of coronary calcification. Therefore, other forms of subclinical atherosclerosis such as development of non-calcified coronary plaques or subclinical atherosclerosis in carotid, aorta and iliac arteries could not be assessed in this study (23, 24, 33). In addition, previous studies have demonstrated that statin therapy may influence coronary plaque calcification (34). However, the retrospective study design did not collect complete history of the lipid-lowering drugs. Further studies are warranted to assess multiterritorial subclinical atherosclerosis in Asian population.

## Conclusion

In summary, we developed and validated successfully a LASSO-derived prediction nomogram based on 10 routine clinical parameters conveniently including “sex,” age,” “hypertension,” “smoking habit,” “GGT,” “CRP,” “HDL-C,” “cholesterol,” “waist circumference,” and “follow-up period,” and demonstrated that it provides a good level of performance for predicting subclinical coronary atherosclerosis in subjects with baseline zero score. This nomogram could help clinicians to identify subclinical coronary atherosclerosis in subjects at low to intermediate risk for guidance for the primary preventive strategies in individual-based preventive cardiology.

## Data Availability Statement

The raw data supporting the conclusions of this article will be made available by the authors, without undue reservation.

## Ethics Statement

The studies involving human participants were reviewed and approved by Institutional review boards of Kaohsiung Veterans General Hospital. Written informed consent for participation was not required for this study in accordance with the national legislation and the institutional requirements. Written informed consent was not obtained from the individual(s) for the publication of any potentially identifiable images or data included in this article.

## Author Contributions

F-ZW prepared the manuscript. All authors contributed to the data collection and analyses, edited the draft manuscript, and approved the final manuscript.

## Conflict of Interest

The authors declare that the research was conducted in the absence of any commercial or financial relationships that could be construed as a potential conflict of interest.

## Abbreviations

CT: computed tomography
CAC: coronary artery calcification
LDL-C: low-density lipoprotein cholesterol
HDL-C: high-density lipoprotein cholesterol
HbA1c: hemoglobin A1c
VCAM: vascular cell adhesion molecule-1
FRS: Framingham risk score
ASCVD: atherosclerotic cardiovascular disease
BMI: Body mass index
SBP: systolic blood pressure
DBP: diastolic blood pressure
CRP: C-reactive protein
GGT: Gamma-Glutamyl Transferase
CTA: CT angiography
SCCT: Society of Cardiovascular Computed Tomography
LASSO: least absolute shrinkage and selection operator
AIC: Akaike information criterion
BIC: Bayesian information criterion
CAD-RADS^TM^: Coronary Artery Disease Reporting and Data System
OR: odds ratio
CVHI: cardiovascular health index
CIMT: carotid intima-media thickness
MetS: metabolic syndrome.
